# The evolution of digital health and its continuing challenges

**DOI:** 10.1186/s44247-022-00004-x

**Published:** 2023-01-24

**Authors:** Alison Cuff

**Affiliations:** BioMed Central, Berlin, Germany

Digital health is transforming medical and health practices. The field has seen rapid growth; the development of new technologies facilitates medical research as well as personalized medicine. Digital health has revolutionized the delivery of healthcare; it is changing the way in which we diagnose, treat, manage and prevent health conditions.

It is a multifaceted discipline involving multiple stakeholders, including clinicians and researchers with expertise in diverse areas, such as healthcare, public health, data technologies, health informatics and biomedical engineering. It will be of no surprise then that current research trends in digital health are diverse and wide ranging. Examples include advances in artificial intelligence leading to improvements in diagnosis and drug development, mHealth apps to support mental health, wearables to assess athletic performance or musculoskeletal dysfunction, various digital health solutions to support the elderly and people with chronic diseases, virtual medically related training, and telehealth.

Digital technology is also a major factor in shifting the focus of healthcare from healthcare professionals to patient-centric. Many digital health tools, particularly wearables and mHealth apps, now place patients in the front seat.

There is no doubt that the covid-19 pandemic has thrust digital health into the limelight thanks to the demand for remote telehealth-based monitoring of covid patients, for example, and smartphone apps to prove vaccination status. However, the increase of digital health technologies comes with the risk of digital inequality. A number of studies have pointed to poor internet access and lower levels of digital literacy amongst particular groups, such as the elderly, certain ethnic minorities or people living in poverty, as being major barriers to digital healthcare access (https://pubs.rsna.org/doi/10.1148/radiol.2020192224).

Digital research is not in itself a new approach. Computational biology has been around since the 1970s (https://www.nature.com/articles/s41746-020-00323-1). The evolution of computer technology and, in particular, computational power, has led to the development of specific computational scientific disciplines such as bioinformatics and systems biology.

Advances in artificial intelligence, machine learning and deep learning have since led to the development of tools that can generate, store and interpret reams of medically related data, create models of biological systems that stimulate disease and the birth of precision medicine. And, as is the case of digital healthcare access, this does come with some significant challenges; this includes questions regarding the quality of data and security concerns.

Patient data is highly sensitive and there are concerns that data anonymization may not be enough to preserve privacy (https://bmjopen.bmj.com/content/11/10/e053440). In any case, collecting, curating and maintaining high quality data is time consuming and expensive. Bias may also be a risk, due to issues around availability of data and the sources used (https://www.ncbi.nlm.nih.gov/pmc/articles/PMC3068925/). Data needs to be devoid of any inbuilt bias that could affect the performance of AI algorithms, with potentially devastating effects.

The take home message is this. Digital health is an exhilarating field that is gathering pace. It has already shown potential to revolutionize the way we think about healthcare and medical research. But there are challenges that can’t be ignored. It is this journey that BMC Digital Health aims to explore, highlighting good quality, sound science in the field that not only highlights the strides in the field but also takes on the issues that remain.

